# Component-resolved diagnosis in adult patients with food-dependent anaphylaxis^☆^

**DOI:** 10.1016/j.waojou.2021.100530

**Published:** 2021-03-12

**Authors:** Pawel Dubiela, Sabine Dölle-Bierke, Stefanie Aurich, Margitta Worm, Karin Hoffmann-Sommergruber

**Affiliations:** a Department of Pathophysiology, Medical University of Vienna, Vienna, Austria; b Department of Regenerative Medicine and Immune Regulation, Medical University of Bialystok, Bialystok, Poland; c Division of Allergology and Immunology, Department of Dermatology, Venereology and Allergology, Charité - Universitätsmedizin Berlin, Corporate Member of Freie Universität Berlin, Humboldt-Universität zu Berlin, And Berlin Institute of Health, Berlin, Germany; d Department of Dermatology, Venereology and Allergology, LICA - Comprehensive Allergy Center, University Hospital, Leipzig, Germany

**Keywords:** Allergy, Component resolved diagnosis, Food anaphylaxis, ImmunoCAP-ISAC, CCD, Cross-reactive carbohydrate determinants, ELISA, Enzyme-Linked Immunosorbent Assay, IgE, Immunoglobulin E, nsLTP, Non-specific lipid transfer protein, OFC, Oral food challenge, PR10, Pathogenesis-related protein family 10, sIgE, Specific immunoglobulin E, SPT, Skin prick test

## Abstract

Food anaphylaxis is a severe, potentially life-threatening, systemic hypersensitivity reaction. Within a retrospective study we applied ImmunoCAP-ISAC in a heterogenous cohort of 54 food anaphylactic patients and compared its performance to conventional *in vitro* (ELISA, ImmunoCAP) and *in vivo* (skin prick test, oral food challenge) diagnosis. Comparing clinical diagnosis with results obtained by ImmunoCAP-ISAC we obtained moderate agreement (kappa 0.524, p < 0.05). The comparison between SPT and ImmunoCAP vs ImmunoCAP-ISAC indicates a good sensitivity of microarray testing. Among the 54 tested sera, 36 and 41 were in substantial agreement with results obtained by SPT (69%, kappa 0.667, p < 0.05) and ImmunoCAP-ISAC (76%, kappa 0.759, p < 0.05), respectively. Within this adult anaphylaxis cohort, plant food allergens were identified as the predominant IgE-binding proteins, with PR10 proteins, ω-5-gliadin and nsLTPs as the most frequent ones. In summary, microarray based IgE testing may help to unravel the elicitating food in anaphylaxis in particular when the elicitor is so far unknown.

Food anaphylaxis is a severe, potentially life-threatening, systemic hypersensitivity reaction, characterized by the rapid onset of serious airway, breathing, or circulatory problems.^1^^,^^2^ The estimated lifetime prevalence of anaphylaxis is 0.3%, with a high probability of being underdiagnosed.^3^ One of the most frequent underlying diseases in anaphylaxis is food allergy. To date, with the exception of peanut, immunotherapy for food allergy is not available. Therefore, the identification of the most frequent elicitors is of utmost importance. In past years, a network of severe allergic reactions has been established to collect standardized data for anaphylactic reactions.^4^ According to the European Academy of Allergy and Clinical Immunology (EAACI) guidelines on food allergy diagnosis, and treatment, the methods of choice for identifying the eliciting food comprise: i) *in vitro* determination of circulating allergen-specific Immunoglobulin E (sIgE), ii) *in vivo* skin prick tests (SPT), and iii) oral food challenges (OFC).^5^ Oral food challenge remains the diagnostic gold standard test for food allergy. However, in clinical practice, there are often logistic barriers to perform food challenges in outpatient settings. Lack of human resources and time are the most frequently listed impediments reported by allergists in an American survey.^6^ Recent data provide evidence that component-resolved diagnosis facilitates a patient-specific sensitization profile, which improves the management of patients with idiopathic anaphylactic reactions.^7^ It could also be useful in the detection of culprit foods especially in cases of cofactor-enhanced food-dependent anaphylaxis.^8^, ^9^, ^10^

The aim of the present study was to apply the allergen microarray-based analysis in a heterogenous cohort of anaphylactic patients and to compare its performance to conventional *in vitro* (ImmunoCAP) and *in vivo* (SPT, OFC) diagnosis. We performed this retrospective study in a cohort of 54 adult patients (mean age 42.7y; range 21–68y) from the Department of Dermatology, Venereology, and Allergology, Charite, Berlin. Inclusion criteria were history of food allergy and the recent report of at least 1 anaphylactic episode. For all subjects a SPT with a predefined standard panel was performed covering the most frequent food allergens. If the suspected food allergen was a plant, a standard panel for inhalant allergens was tested as well to unravel the possibility of pollen associated food allergy. Skin prick test was performed with fresh foods as prick to prick (ie, celery, gluten) and commercial extracts (ie, peanut, inhalant allergens). Total and sIgE levels (ImmunoCAP) were determined. Twenty-nine individuals underwent double-blind, placebo-controlled OFCs. Furthermore, all patients' sera were tested by ImmunoCAP-ISAC (e) 112 Multiplex Phadia (Thermofisher) following the manufacturer's instructions. The diagnostic approaches were compared using Kappa statistics based on GraphPad Prism 7 for Windows (including n ≥ 4 samples). Kappa values < 0.2 indicate poor agreement; 0.21 to 0.40: fair agreement; 0.41 to 0.6: moderate agreement; 0.61 to 0.80 substantial agreement; and 0.81 to 1.00: almost total agreement.^11^

## Patients’ characteristics and diagnosis of food allergy

All subjects experienced an acute systemic severe allergic reaction with symptoms of the respiratory tract and/or the cardiovascular system^12^ and were referred to the Department of Dermatology, Venereology and Allergology, Charite, Berlin for further evaluation. The clinical center followed the national and international diagnostic algorithm for food allergy comprising case history, sIgE/SPT, OFC.^2^^,^^13^ Oral food challenge was offered to all patients. However, OFC is not performed if patients do not give their consent or if any contraindications are present (eg, intake of beta-blocker). Based on the diagnostic workup, 40 out of 54 cases were caused by plant food allergens and 2 cases displayed reactions against seafood (calamari and shrimp), and in 12 patients the eliciting food could not be identified.

**Fig. 1:**
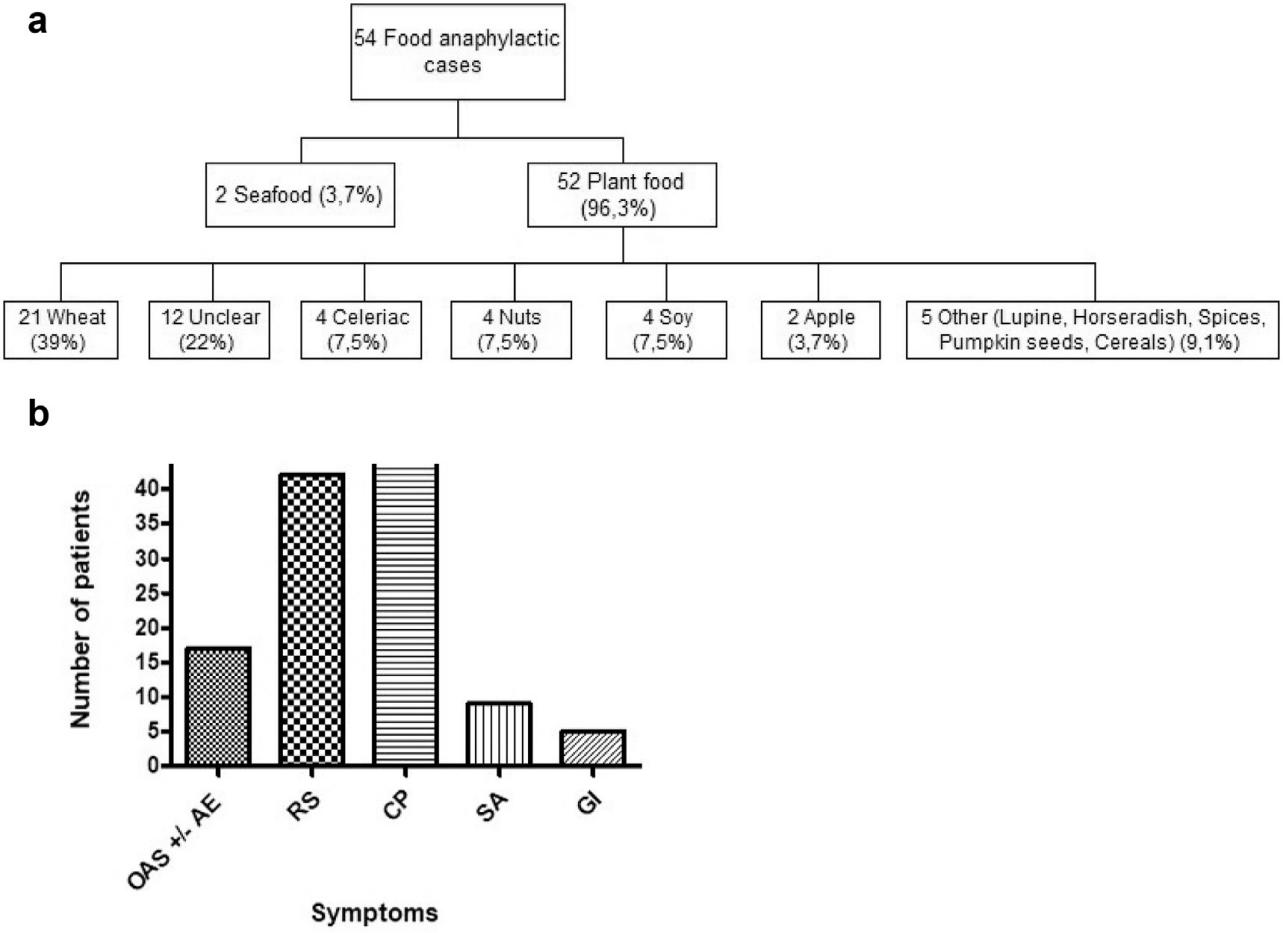
**Elicitors (A) and symptoms (B) of anaphylactic reactions in a cohort of 54 food allergic patients**. OAS – Oral allergy syndrome, AE – Angioedema, RS – Respiratory symptoms, CP – Circulation problems, SS – Skin symptoms (Erythema, pruritus, urticaria), GI – Gastrointestinal tract problems

Among the plant food elicitors, wheat was by far the most frequently identified source (n = 21 of IgE sensitization). Further elicitors were celery (n = 4), tree nuts (n = 4), soy (n = 4), apple (n = 2), and lupine, horseradish, spices, pumpkin seeds, and cereals (n = 1 each) (Fig. 1 A). The majority of the patients suffered from circulatory symptoms (n = 44), respiratory difficulties (n = 42), angioedema, and erythema (n = 17). Off note, gastrointestinal symptoms were reported in only 5 cases (Fig. 1 B).

## Clinical diagnosis of food allergy versus ImmunoCAP-ISAC

**Fig. 2:**
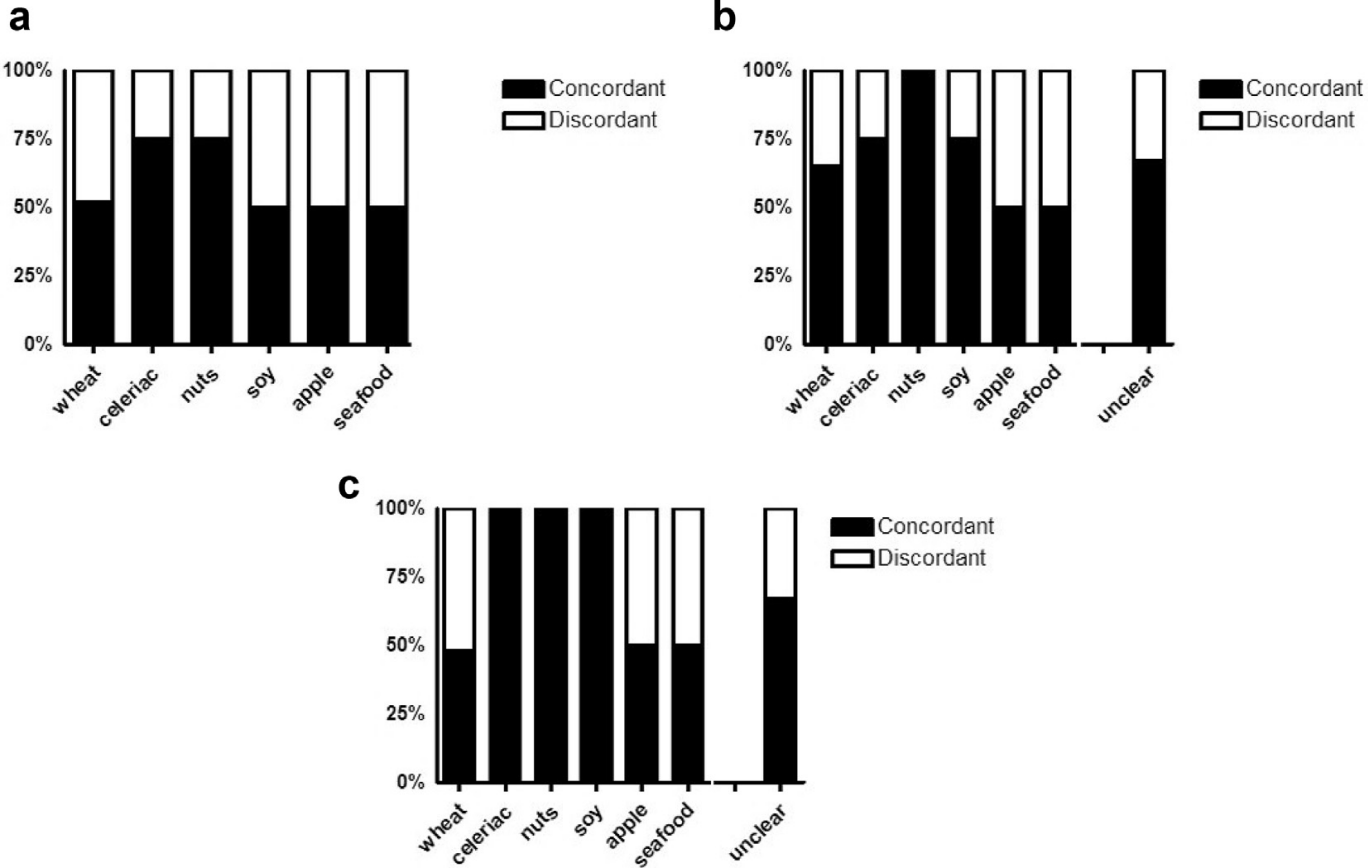
**Comparison of clinical diagnosis *versus* ImmunoCAP-ISAC (A); SPT *versus* ImmunoCAP-ISAC (B); and ImmunoCAP versus ImmunoCAP**-**ISAC (C)**. Total of 54 adult patients with food anaphylactic reactions were tested by SPT, and sIgE determined by ImmunoCAP and ImmunoCAP-ISAC, respectively. Concordant and discordant results are presented with black and white bars, respectively. Other represents - lupine, horseradish, spices, pumpkin seeds and cereals. Unclear (n = 12) cases were not included in comparison between clinical diagnosis versus ImmunoCAP-ISAC (A)

The comparison of clinical diagnosis (as described above) and results obtained by ImmunoCAP-ISAC were in moderate agreement (52%, kappa 0.524, p < 0.05). The highest sensitivity of the microarray was observed for celeriac as well as for tree nuts (75%, kappa 0.550, p < 0.05). IgE binding to wheat (52%, kappa 0.524, p < 0.05), soy, apple, and seafood was detected with medium sensitivity (50%; Fig. 2 A). Patients that suffered from yet unknown food source (not included in statistical comparison with ImmunoCAP-ISAC) revealed no IgE binding on the chip (n = 7) or unclear profile with reaction to PR-10 (n = 4) allergens or CCD (n = 1). Interestingly, specific testing for Tri a 19 using an in-house ELISA, resulted in 4 additional sera positive for Tri a 19 from patients suffering from co-factor dependent wheat anaphylaxis, thus increasing the sensitivity of *in vitro* testing to 71% (data not shown).

## SPT versus ImmunoCAP-ISAC

Results obtained by SPT and ImmunoCAP-ISAC showed substantial sensitivity of the microarray test. Among the 54 tested sera, 36 were in good agreement with results obtained by SPT (69%, kappa 0.667, p < 0.05). The highest percentage of correlation was for tree nuts (walnut, hazelnut and cashew; 100%, kappa 1.000, p < 0.05) followed by celeriac and soy (75%, kappa 0.550, p < 0.05), unclear elicitors (67%, kappa 0.667, p < 0.05), and wheat (67%, kappa 0.667, p < 0.05). Slightly lower sensitivity was observed for apple and seafood (50% each, Fig. 2 B).

## ImmunoCAP versus ImmunoCAP-ISAC

When comparing the results obtained from ImmunoCAP versus ImunoCAP-ISAC, agreements for both tests were obtained in 76% (kappa 0.759, p < 0.05); 41 serum samples out of 54 (Fig. 2 C). As for SPT, the highest percentage of correlation was found for tree nuts (walnut, hazelnut, and cashew), celeriac, and soy (100%, kappa 1.000, p < 0.05) followed by unclear elicitors (67%, kappa 0.667, p < 0.05), and seafood (50%). Surprisingly, the lowest correlation was observed for wheat allergy (48%, kappa 0.476, p < 0.05) (Fig. 2 C). However, if results obtained by an in-house ELISA were included 4 additional sera positive to Tri a 19, the concordance would reach 67% (data not shown).

## ImmunoCAP-ISAC

Based on the ImmunoCAP-ISAC data, most of the patients (n = 35) were sensitized to more than 1 allergen, including inhalant allergen sources, whereas 6 patients displayed a mono-sensitization. Thirty sera tested on ImmunoCAP-ISAC revealed sIgE directed against food allergens and 2 sera were exclusively sensitized to CCD. In 22 sera no sIgE with reactivity to any food allergen present on the ImmunoCAP-ISAC was detected. Out of those patients 8 patients had wheat allergy, 2 patients had anaphylaxis to soy, and 1 to each of the following: apple, cashew, celery, horseradish, and seafood. Moreover, for 7 patients’ sera the elicitor was unknown. This may be due to either low test sensitivity or else it indicates that relevant food allergens available for testing are still lacking.

PR10 was identifed as the leading protein family being recognized by sIgE from 19 sera followed by ω-5-gliadin from wheat (n = 8), and non-specific lipid transfer proteins (nsLTPs; n = 5). So far, severe – anaphylactic reactions to PR10 and nsLTP are regarded as rather uncommon. However, especially in co-factor enhanced food allergic reactions, ns-LTPs and PR10 have been described as causative allergens.^9^^,^^10^

Concomitant sensitizations to inhalant allergens were determined for grass pollen (n = 25), Fagales pollen (n = 19), animal dander (n = 16), weed pollen (n = 15), olive pollen (n = 9), fungal spores (n = 8), and mites (n = 7). These results were in substantial agreement with SPT (76%; concordance in 75 out of 99 results, kappa 0.758, p < 0.05).

In summary, microarray-based IgE testing was applied to obtain an allergen-based sensitization profile of a group of patients who had experienced anaphylactic episodes. This group of patients was quite heterogeneous regarding their causative foods. Therefore, the outcome and benefit of applying ImmunoCAP-ISAC varies and mostly depends on the causative foods. Based on our results, the microarray proved superior in the case of tree nuts (walnut, hazelnut, and cashew), soy, apple, and seafood allergy. Furthermore, it provided insight into the prediction of wheat anaphylaxis. However, it has to be mentioned that sensitivity of the test regarding Tri a 19 needs to be increased, since wheat is a relevant food source inducing severe allergic reactions also caused by additional allergens. Within this cohort, plant food allergens were identified as the predominant IgE-binding proteins, with PR10 proteins, ω-5-gliadin and nsLTPs as the most frequent ones.

In general, results obtained by microarray were in moderate to substantial agreement with the clinical diagnosis and provided additional information on concomitant sensitizations. Better agreement was observed when correlating data from ImmunoCAP-ISAC with ImmunoCAP and SPT, respectively, since all these tests provide evidence of allergic sensitization.

To date, the allergen panel provided on the ImmunoCAP-ISAC is the most complete commercially available diagnostic tool allowing simultaneous testing of 112 allergens with a minimum amount of 30 μL of serum.

However, considering the relatively high number of negative outcomes within our study indicates the necessity to improve this test format further with regard to sensitivity and extension/completion of the food allergen panels. Especially, there is a need to provide additional molecules for wheat and seeds. In summary, the microarray based IgE testing provides helpful information on the sensitization pattern. Although relevant allergens predictive for severe allergic reactions are still lacking this approach seems to be promising to contributing to a better management of the patient in fine tuning dietary recommendations and avoidance strategies.

## Financial support

Supported by the grants “Wirtschaftskammerpreis an der Medizinischen Universität Wien“, SFB F4603 (Austrian Science Fund) to K. Hoffmann-Sommergruber and W1248 (Austrian Science Fund) to P. Dubiela.

## Consent for publication

All authors consented for publication in this study. Authors confirm that the manuscript is original, has not been published before, is not currently being considered for publication elsewhere, and has not been posted to a preprint server.

## Statement of contribution

MW and KHS conceived the study design, PD, SDB, SA acquire data and performed the experiments, all the authors contribute to the data analysis and actively participated in the manuscript writing.

## Availability of data and materials

Contact the corresponding author for questions regarding data and materials.

## Ethics statement

Informed written consent was obtained from all participants, and the use of serum samples for this study was approved by the ethics committee of the Medical University of Vienna (No. 2196/2016). Authors ensure following all ethical publication practices involving transparency and integrity in the publication of the manuscript.

## Acceptance of editorial policy

Authors confirm acceptance of editorial policy.

## Declaration of competing interest

Dr. Hoffmann-Sommergruber reports grants from Wirtschaftskammerpreis an der Medizinischen Universität Wien, grants from Austrian Science Funds SFB F4603, during the conduct of the study as potential conflicts of interest according to the ICMJE: The Work Under Consideration for Publication. Dr Worm and Dr Dubiela disclose other potential conflict of interest according to the ICMJE: Relevant Financial Activities Outside the Submitted Work.
